# SARS-CoV-2 Causes Mitochondrial Dysfunction and Mitophagy Impairment

**DOI:** 10.3389/fmicb.2021.780768

**Published:** 2022-01-06

**Authors:** Chao Shang, Zirui Liu, Yilong Zhu, Jing Lu, Chenchen Ge, Cuiling Zhang, Nan Li, Ningyi Jin, Yiquan Li, Mingyao Tian, Xiao Li

**Affiliations:** ^1^Changchun Veterinary Research Institute, Chinese Academy of Agricultural Sciences (CASS), Changchun, China; ^2^College of Veterinary Medicine, Jilin University, Changchun, China; ^3^Academician Workstation of Jilin Province, Changchun University of Chinese Medicine, Changchun, China; ^4^Agricultural College, Yanbian University, Yanji, China; ^5^Jiangsu Co-innovation Center for Prevention and Control of Important Animal Infectious Diseases and Zoonoses, Yangzhou, China

**Keywords:** SARS-CoV-2, mitochondria, mitophagy, Tom20, viral RNA localization

## Abstract

Mitochondria, which is essential for adequate innate immune response, energy metabolism and mitochondria reactive oxygen species (ROS) production, might be in the cross fire of Severe acute respiratory syndrome coronavirus-2 (SARS-CoV-2) and host cell defense. However, little is known about interactions between mitochondria and SARS-CoV-2. We performed fluorescent microscopy and found an enrichment of SARS-CoV-2 replication products double stranded RNA (dsRNA) within mitochondria. The entry process of dsRNA might be mediated by Tom20 as observed by reduced mitochondrial localization of SARS-CoV-2 dsRNA in Tom20 knockdown cells. Importantly, decreased mitochondrial localization of dsRNA, as well as mitochondrial membrane stabilizers mdivi-1 and cyclosporin A, inhibited viral load in cells. Next, we detected mitochondrial dysfunction caused by SARS-CoV-2 infection, including mitochondrial membrane depolarization, mitochondrial permeability transition pore opening and increased ROS release. In response to mitochondrial damage, we observed an increase in expression and mitochondrial accumulation of Pink1 and Parkin proteins, as well as Pink-1-mediated recruitment of P62 to mitochondria, suggesting initiated mitophagy for mitochondrial quality control and virus clearance. Nevertheless, we observed that mitophagy was inhibited and stayed in early stage with an unchanged Hsp60 expression post SARS-CoV-2 infection. This might be one of the anti-autophagy strategies of SARS-CoV-2 and we used co-immunoprecipitation to found that SARS-CoV-2 infection inhibited P62 and LC3 binding which plays a critical role in selective envelopment of substrates into autophagosomes. Our results suggest that mitochondria are closely involved in SARS-CoV-2 replication and mitochondrial homeostasis is disrupted by SARS-CoV-2 in the virus-cell confrontation.

## HIGHLIGHTS

- An RNA-GPS study that compared hundreds of SARS-CoV-2 genomes to the human transcriptome predicts the SARS-CoV-2 RNA genome localization in the host mitochondrial matrix.
- However, little is known about the effect of SARS-CoV-2 on mitochondrial homeostasis.
- Here, we demonstrate that mitochondria are closely involved in SARS-CoV-2 replication and Tom20 plays a vital role in the entry of viral RNA into mitochondria.
- Moreover, our study gives insights into the confronting actions between SARS-CoV-2 and host cell defensive activities in mitochondria.
- We demonstrate that SARS-CoV-2 induces mitochondrial damage of mitochondrial membrane depolarization, mitochondrial permeability transition pore opening and increased ROS release.
- Concomitantly, mitophagy is initiated through Pink1/Parkin pathway by host cell for mitochondrial quality control.
- However, SARS-CoV-2 inhibits P62 and LC3 binding which plays a critical role in selective envelopment of mitochondria into autophagosomes.
- The present study increases our understanding of SARS-CoV-2-mitochondria interactions and assists the development of effective interventions targeting mitochondria.

## Introduction

Coronavirus Disease 2019 emerged in December 2019 and quickly escalated into a global pandemic with more than 124 million confirmed cases by the end of March 2021 [World Health Organization (WHO), 2020; Zhou et al., 2020, December 30]. The causative agent, Severe acute respiratory syndrome coronavirus-2 (SARS-CoV-2), is a positive sense, single strand RNA (+ RNA) virus and belongs to the genus of β coronavirus (Kim et al., 2020; Zhu et al., 2020).

+RNA viruses target on host intracellular membrane structures as sites for RNA synthesis, for example, mitochondria for Flock house virus, lysosome for Semliki forest virus and modified membrane structures from endoplasmic reticulum for Equine arteritis virus and Severe acute respiratory syndrome coronavirus (SARS-CoV) (Salonen et al., 2005; Knoops et al., 2008, 2012; Miller and Krijnse-Locker, 2008). In β coronavirus, including SARS-CoV and mouse hepatitis virus, double membrane vesicles (DMV) originated from ER is the most abundant components of viral replication organelles in host cell for viral RNA synthesis (Gosert et al., 2002; Knoops et al., 2008; Reggiori et al., 2010). Similarly, SARS-CoV-2 induces DMV formation and the replication of SARS-CoV-2 is believed to mainly rely on DMV structures (Wolff et al., 2020). However, little is known if mitochondria are associated with SARS-CoV-2 replication. An RNA-GPS study that compared hundreds of SARS-CoV-2 genomes to the human transcriptome predicts the SARS-CoV-2 RNA genome and sgRNAs to be enriched toward the host mitochondrial matrix and nucleolus (Wu et al., 2020).

Insight into how SARS-CoV-2 interacts with host cell defense machinery represents a significant research topic. Mitochondria, which is essential for adequate innate immune response, energy metabolism and mitochondria reactive oxygen species (ROS) production, might be in the cross fire of SARS-CoV-2 and host cell defense (Burtscher et al., 2020). For host cell defense, autophagy represents a powerful tool against virus in which the double membrane vesicles termed autophagosomes may deliver trapped viral cargos and damaged organelles to lysosome for viral clearance (Kuballa et al., 2012; Choi et al., 2018). In mitophagy, a specific form of autophagy, flawed mitochondria were selectively sequestered into autophagosomes and subsequently degraded in lysosomes for mitochondria quality control (Lemasters, 2005; Ashrafi and Schwarz, 2013). Faced with the ongoing evolutionary arms race, certain viruses, including polioviruses, foot-and-mouth disease virus, SARS-CoV and dengue virus, have adopted various strategies to interrupt, escape or manipulate multiple steps during autophagy and promote viral replication (Dales et al., 1965; Prentice et al., 2004; Miller et al., 2007; Choi et al., 2018; Shojaei et al., 2020). Until now, the interaction of SARS-CoV-2 with cellular autophagy machinery is only beginning to be understood and no studies in SARS-CoV-2-related mitophagy has been reported.

Here, we examined the subcellular localization of double-stranded RNA (dsRNA) intermediates of + RNA virus replication and observed a remarkable enrichment of dsRNA signals colocalization within mitochondria. Next, we detected mitochondrial lesion caused by SARS-CoV-2 infection and the accumulation of DMV structures among the damaged mitochondria, and found the antiviral replication activity of mitochondria membrane stabilizer which can improve mitochondria abnormity. Thus, we demonstrate that the mitochondria are closely involved in SARS-CoV-2 replication. Moreover, we reported a role of mitochondrial outer membrane protein Tom20 in the mitochondrial localization of SARS-CoV-2 RNA. We speculate that the mitochondrial damage caused by SARS-CoV-2 may subsequently activate the classic Pink1/Parkin pathway associated with mitophagy for clearance of and defense against virus. However, despite activated Pink1/Parkin pathway and mitochondrial P62 accumulation that marks damaged mitochondria as substrates of autophagosome, we observed that mitophagy was inhibited and stayed in early stage. This may be one of the anti-autophagy strategies of SARS-CoV-2 and we used co-immunoprecipitation to found that SARS-CoV-2 infection inhibited P62 and LC3 binding which plays a critical role in selective envelopment of substrates into autophagosomes. Our results give new understandings to confronting actions of SARS-CoV-2 and host cell defensive activities in mitochondria.

## Results

### SARS-CoV-2 dsRNA Localizes in Mitochondria

**FIGURE 1:**
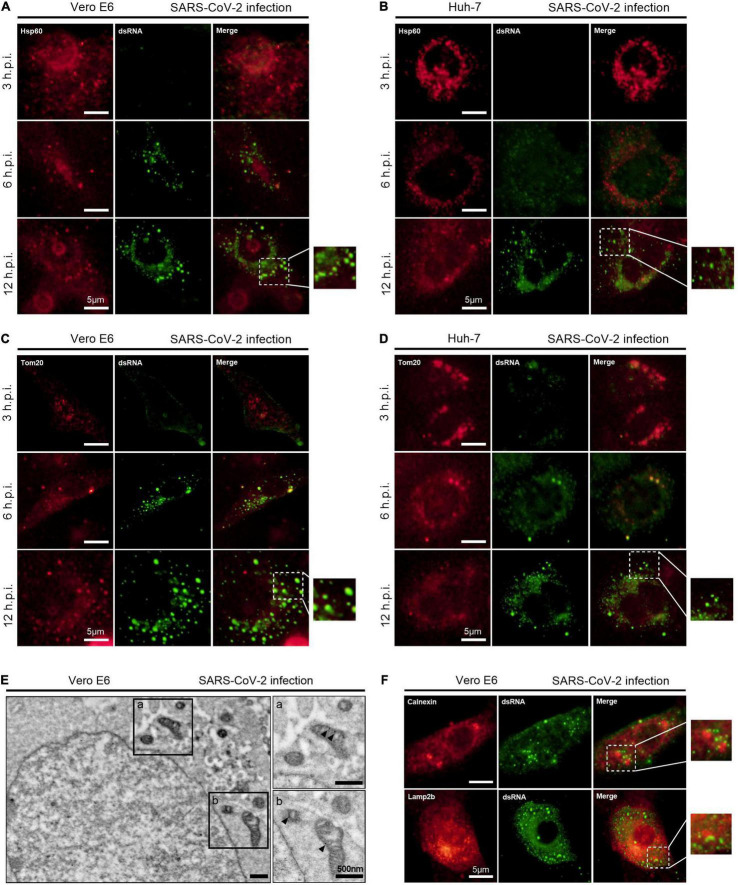
SARS-CoV-2 dsRNA localizes in mitochondria. **(A,B)** Vero E6 and Huh-7 cells were fixed and visualized by fluorescence microscopy using antibodies against Hsp60 (red) and dsRNA (green) at 3, 6, and 12 h post infection (h.p.i.). **(C,D)** Vero E6 and Huh-7 cells were infected with SARS-CoV-2 for 3, 6, and 12 h and visualized by fluorescence microscopy with antibodies recognizing Tom20 (red) and dsRNA (green). **(E)** Vero E6 cells were visualized by immunoelectron microscopy using antibodies against dsRNA at 12 h post infection. **(F)** Vero E6 cells were visualized by fluorescence microscopy using antibodies against Calnexin (red), Lamp2b (red) and dsRNA (green) at 12 h post SARS-CoV-2 infection. White scale bars = 5 μm; black scale bars = 500 nm.

dsRNA is the intermediate products of + RNA virus replication (Knoops et al., 2008, 2012). To find some clues for the role of mitochondria in SARS-CoV-2 replication, we immunofluorescently stained SARS-CoV-2-infected cells using antibodies specific for dsRNA and the mitochondrial markers, including the mitochondrial matrix protein HSP60 and the outer mitochondrial membrane protein Tom20 (Gupta and Knowlton, 2005). In both Vero E6 and Huh-7 cells, a merge of signals from Hsp60 and dsRNA were observed at 12 h post SARS-CoV-2 infection (Figures 1A,B). Similarly, colocalization of Tom20 and dsRNA was also apparent at 6 and 12 h post infection (Figures 1C,D). We next performed immunoelectron microscopy experiments using Vero E6 cells to further confirm the mitochondrial localization of SARS-CoV-2 dsRNA directly. In mitochondria, small numbers of gold particle of dsRNA signals were visualized at 12 h post infection (Figure 1E), suggesting dsRNA accumulation and possible virus replication in mitochondria.

Moreover, we evaluated the dsRNA localization in other primary subcellular organelles, including endoplasmic reticulum and lysosomes. No overlapping signals of dsRNA and the endoplasmic reticulum marker Calnexin, as well as dsRNA and the lysosome marker Lamp2b, were observed in Vero E6 cells at 12 h post SARS-CoV-2 infection (Figure 1F).

### SARS-CoV-2 Induces Mitochondrial Dysfunction

**FIGURE 2:**
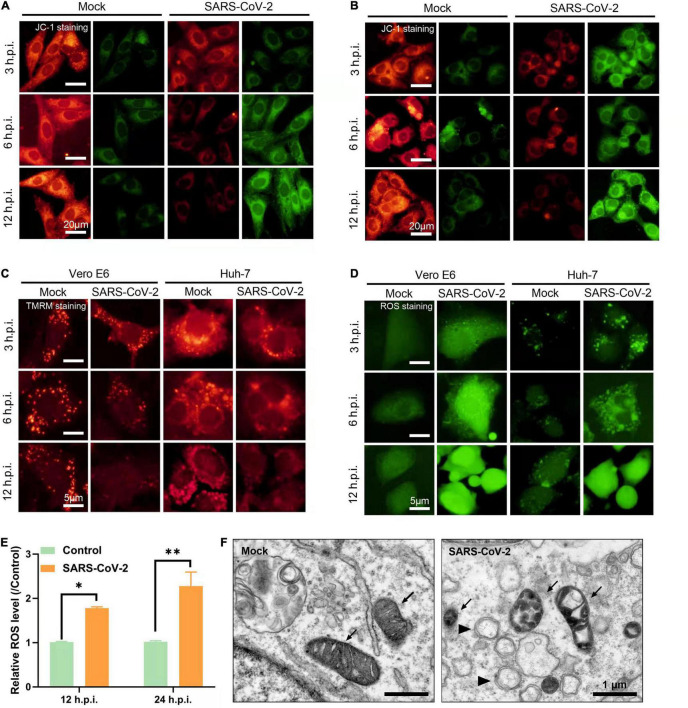
SARS-CoV-2 induces mitochondrial dysfunction. **(A,B)** Vero E6 **(A)** and Huh-7 cells **(B)** were stained with JC-1 at 3, 6, and 12 h post infection and visualized by fluorescence microscopy showing mitochondrial membrane depolarization induced by SARS-CoV-2. **(C)** Vero E6 and Huh-7 cells were stained with TMRM at 3, 6, and 12 h post infection and visualized by fluorescence microscopy showing mitochondrial permeability transition pore (MPTP) opening induced by SARS-CoV-2. **(D)** Vero E6 and Huh-7 cells were stained with carboxy-H2DCFDA at 3, 6, and 12 h post infection and visualized by fluorescence microscopy showing reactive oxygen species (ROS) induced by SARS-CoV-2. **(E)** Flow cytometry analysis of ROS in Vero E6 cells infected with SARS-CoV-2 at 12 and 24 h. **(F)** Vero E6 cells were fixed and visualized by transmission electron microscopy at 24 h post SARS-CoV-2 infection. Black arrow points to mitochondria and black arrow head points to viral replication complexes. White scale bars = 5 μm; black scale bars = 1 μm. *p < 0.05, **p < 0.01.

To examine the effect of SARS-CoV-2 on mitochondrial function, we performed JC-1 staining to detect mitochondrial membrane potential (ΔΨm) on Vero E6 and Huh-7 cells. JC-1 dye aggregates in healthy mitochondria and fluoresces red. Upon the loss of ΔΨm, JC-1 can only exists as monomers and fluoresces green (Koppikar et al., 2010). In Vero E6 and Huh-7 cells, we found that SARS-CoV-2 disrupted the ΔΨm as observed by decrease in the intensity of red fluorescence, as well as increase in JC-1 monomers (green fluorescence) along with time (Figures 2A,B).

Depolarization of ΔΨm will lead to the opening of mitochondrial permeability transition pore (MPTP) and the change of mitochondrial permeability. Next, we used TMRM dye to detect MPTP opening. TMRM dye is lipophilic and has strong red fluorescence in the inner membrane of mitochondria. Upon MPTP opening, the dye enters the cytoplasm resulting into decrease in red fluorescence (Javed et al., 2012; Boyman et al., 2019). In accordance with this, the red fluorescence of TMRM gradually decreased at 3, 6, and 12 h after SARS-CoV-2 infection in Vero E6 and Huh-7 cells, suggesting the MPTP opening and the release of mitochondrial contents into the cytoplasm (Figure 2C).

Mitochondrial respiratory chain and mitochondrial lipid peroxidation are two main sources of ROS production in animal cells. Excessive release of ROS leads to oxidative damage and abnormal energy metabolism (Zorov et al., 2014). We used carboxy-H2DCFDA probe which releases green fluorescence in response to esterase oxidation and can reflect ROS levels by fluorescence intensity (Wu and Yotnda, 2011). By fluorescence microscopy, we observed that the cells infected with SARS-CoV-2 exhibited a time dependent increase in green fluorescence compared to mock-infected cells (Figures 2D,E). This indicates increased ROS release caused by SARS-CoV-2 which might be associated with mitochondrial dysfunction.

Next, we visualized mitochondrial morphology by transmission electron microscopy and found mitochondrial swelling and vacuolization at 24 h in SARS-CoV-2-infected Vero E6 cells. In addition, we observed the virus replication complexes of DMV structures around mitochondria (Figure 2F). Taken together, all these results suggested that SARS-CoV-2 induced mitochondrial dysfunction, including the loss of ΔΨm, MPTP opening and increased ROS release.

### Mitochondrial Depolarization and Mitochondrial Permeability Transition Pore Opening Promote SARS-CoV-2 Replication

**FIGURE 3:**
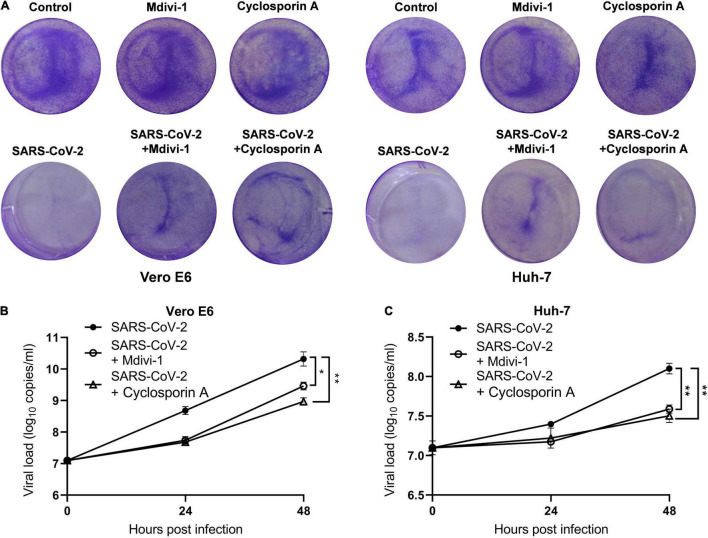
Mitochondrial depolarization and MPTP opening promote SARS-CoV-2 replication. **(A)** Mdivi-1 (5 μM) or cyclosporin A (40 μM) were pretreated 2 h before SARS-CoV-2 infection and crystal violet staining was performed at 48 h. Viral load in SARS-CoV-2-infected Vero E6 **(B)** and Huh-7 cells **(C)**. Mdivi-1 (5 μM) or cyclosporin A (40 μM) were pre-treated 2 h prior to SARS-CoV-2 infection. Data were expressed as mean ± SEM from three independent experiments. *p < 0.05, **p < 0.01.

To investigate the role of mitochondria in SARS-CoV-2 replication, we pretreated cells with mitochondrial membrane stabilizer prior to SARS-CoV-2 infection. Mdivi-1, an inhibitor of mitochondrial fission protein dynamin-related protein-1, can mitigate mitochondrial depolarization and improve the morphological abnormality of mitochondria. First, we analyzed the cytotoxic effects of the two inhibitors in Vero E6 and Huh-7 cells, and found that mdivi-1 (5 μM) and cyclosporin A (40 μM) did not affect cell viability (Figure 3A). In both Vero E6 and Huh-7 cells, mdivi-1 (5 μM) reduced SARS-CoV-2 gene copies at 24 and 48 h post infection. Similarly, cyclosporin A (40 μM), a cyclophilin D inhibitor known to restrain MPTP opening, also decreased SARS-CoV-2 viral load (Figures 3B,C). These results indicates that the loss of ΔΨm and MPTP opening are beneficial to SARS-CoV-2 replication.

### Tom20 Facilitates SARS-CoV-2 dsRNA Localization in Mitochondria

**FIGURE 4:**
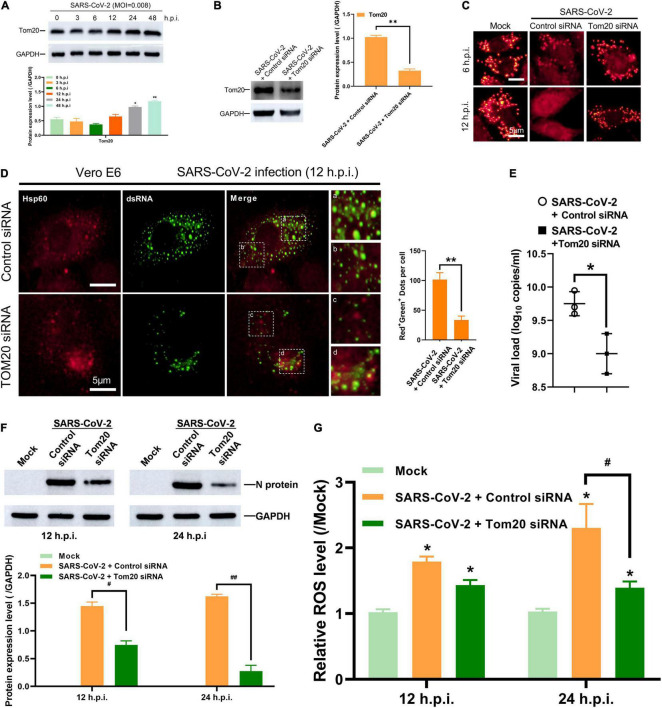
Tom20 facilitates SARS-CoV-2 dsRNA localization in mitochondria. **(A)** Western blotting analysis showing Tom20 expression at 3, 6, 12, 24 and 48 h post SARS-CoV-2 infection in Vero E6 cells. **(B)** Western blotting analysis showing knockdown efficiency of Tom20 siRNA in Vero E6 cells. **(C)** Vero E6 cells transfected with control or Tom20 siRNA were stained with TMRM at 6 and 12 h post infection showing MPTP opening. **(D)** Vero E6 cells transfected with control or Tom20 siRNA were visualized by fluorescence microscopy using antibodies against Hsp60 (red) and dsRNA (green) at 12 h post SARS-CoV-2 infection. The amounts of dots with overlapping red and green fluorescence per cell were calculated from at least 30 cells from each group. White scale bars = 5 μm. **(E)** Viral load at 48 h in Vero E6 cells transfected with control or Tom20 siRNA. **(F)** Western blotting analysis showing N protein expression at 3, 6, 12, and 24 h post SARS-CoV-2 infection in Vero E6 cells transfected with control or Tom20 siRNA. **(G)** Vero E6 cells transfected with control or Tom20 siRNA were infected with SARS-CoV-2 to detect cellular reactive oxygen species at 12 and 24 h. Data were expressed as mean ± SEM from three independent experiments. *p < 0.05, ^**^p < 0.01, ^#^*p* < 0.05, ^##^*p* < 0.01.

The mitochondrial outer membrane protein Tom20 is a subunit of Tom complexes and serves as the central entry gate for nuclear-encoded mitochondrial precursor proteins. Additionally, Tom20 is known to interact with the mitochondrial targeting sequence of many mRNA, and its deletion results in decreases in the localization of the mitochondrial targeting sequence-GFP reporter mRNA (Eliyahu et al., 2010; Lesnik et al., 2015). In Vero E6 cells post SARS-CoV-2 infection, the expression of Tom20 increased gradually along with time (Figure 4A). In order to explore whether Tom20 protein is involved in the mitochondrial entry process of SARS-CoV-2 dsRNA, we knocked down the expression of Tom20 protein by siRNA (Figure 4B). TMRM staining showed that Tom20 knockdown improved mitochondrial permeability at 6 and 12 h post SARS-CoV-2 infection (Figure 4C). This may be related to decreased mitochondrial localization of SARS-CoV-2 dsRNA in mitochondrial as observed by reduced overlapping signals from dsRNA and Hsp60 (Figure 4D), indicating a role of Tom20 in facilitating the mitochondrial entry of SARS-CoV-2 dsRNA. Importantly, this mitochondrial localization is associated to SARS-CoV-2 replication and Tom20 knockdown reduced viral load at 48 h post SARS-CoV-2 infection (Figure 4E). Then, after infection of the tom20-silenced cells with the SARS-CoV-2, it was found that the level of viral N protein (nucleocapsid protein) in the mitochondria was significantly reduced (Figure 4F), and the release of ROS was also significantly reduced (Figure 4G).

### Mitophagy Is Activated *via* Pink1/Parkin Pathway in SARS-CoV-2-Infected Cells

**FIGURE 5:**
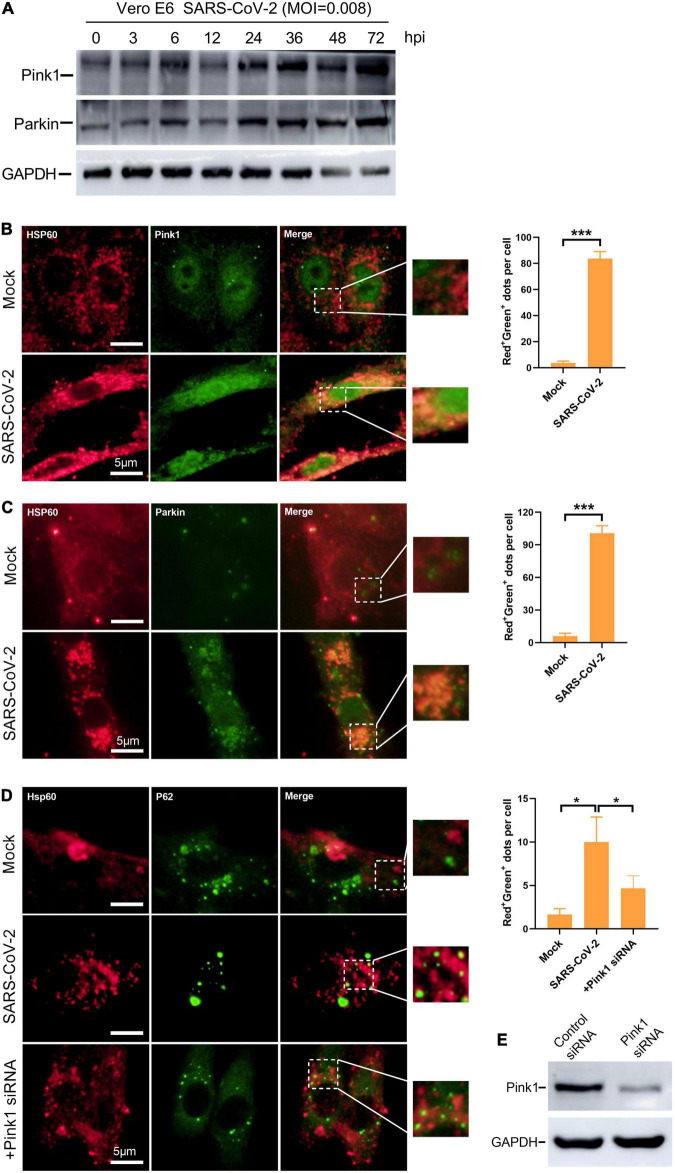
Mitophagy is activated *via* Pink1/Parkin pathway in SARS-CoV-2-infected cells. **(A)** Western blotting analysis showing Pink1 and Parkin expression at 3, 6, 12, 24, 48, and 72 h post SARS-CoV-2 infection in Vero E6 cells. **(B)** Vero E6 cells were mock- or SARS-CoV-2-infected for 12 h and visualized by fluorescence microscopy with antibodies recognizing Hsp60 (red) and Pink1 (green). **(C)** Vero E6 cells were visualized by fluorescence microscopy using antibodies against Hsp60 (red) and Parkin (green) at 12 h post mock or SARS-CoV-2 infection. **(D)** Vero E6 cells were transfected with control or Pink1 siRNA and visualized by fluorescence microscopy using antibodies against Hsp60 (red) and Parkin (green) at 12 h post mock or SARS-CoV-2 infection. **(E)** Western blotting analysis showing knockdown efficiency of Tom20 siRNA in Vero E6 cells. The amounts of dots with overlapping red and green fluorescence per cell were calculated from at least 30 cells from each group. White scale bars = 5 μm. Data were expressed as mean ± SEM from three independent experiments. *p < 0.05, ***p < 0.001.

In defense against SARS-CoV-2, mitophagy represents a powerful tool in mitochondrial quality control and the clearance of viral dsRNA. To further investigate whether the mitochondrial damage induced by SARS-CoV-2 would subsequently initiate mitophagy, we examined the Pink1/Parkin pathway. Under healthy and steady state, Pink1, a mitochondrial serine/threonine kinase, can be rapidly degraded by ΔΨm-dependent proteolytic enzymes. When mitochondrial damage and depolarization occurs, Pink1 accumulates in the outer membrane of mitochondria and recruits Parkin protein (Ashrafi and Schwarz, 2013; Matsuda et al., 2013). Parkin is a cytoplasmic protein with E3 ubiquitin ligase activity. The polyubiquitination of mitochondrial substrate mediated by Parkin is considered to be an important step in inducing mitophagy (Geisler et al., 2010). In SARS-CoV-2-infected Vero E6 cells, the expression of Pink and Parkin increased gradually along with time (Figure 5A). Additionally, by immunofluorescence microscopy, we observed a significant increase in a merge of signals from Hsp60 and Pink1, as well as Hsp60 and Parkin, in SARS-CoV-2 infected Vero E6 cells at 12 h compared to mock-infected cells (Figures 5B,C). These results indicate the activation of Pink1/Parkin pathway and accumulation of Pink1 and Parkin protein in mitochondria by SARS-CoV-2 infection.

Parkin mediates polyubiquitination of mitochondrial substrates which can be recognized by the ubiquitin adaptor protein P62. The recruitment of p62 to mitochondria labeled damaged mitochondria as a selective autophagy substrate (Geisler et al., 2010). In Vero E6 cells, the accumulation of P62 to mitochondria by SARS-CoV-2 infection was confirmed as observed by a substantial increase in the overlapping signals from Hsp60 and P62 (Figure 5D). Next, to explore whether the mitochondrial recruitment of P62 is mediated by Pink1/Parkin pathway, we knocked down the expression of Pink1 protein by siRNA (Figure 5E). In SARS-CoV-2-infected cells, we found decreased mitochondrial localization of P62 by Pink1 knock down (Figure 5D). Taken together, these results suggest the activation of Pink1-Parkin-P62 pathway known for mitophagy in SASRS-CoV-2 infected cells.

### SARS-CoV-2 Restrains Mitophagy *via* Inhibiting P62 and LC3 Binding

**FIGURE 6:**
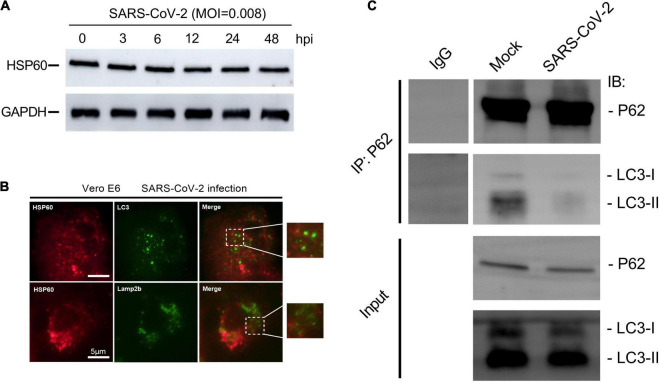
SARS-CoV-2 restrains mitophagy *via* inhibiting P62 and LC3 binding. **(A)** Western blotting analysis showing Hsp60 expression at 3, 6, 12, 24, and 48 h post SARS-CoV-2 infection in Vero E6 cells. **(B)** Vero E6 cells were visualized by fluorescence microscopy using antibodies against Hsp60 (red) and LC3 (green) or Lamp2b (green) at 12 h post SARS-CoV-2 infection. White scale bars = 50 μm. **(C)** Co-immunoprecipitation assays showing levels of LC3 precipitated by P62 in Vero E6 cells at 12 h post mock or SARS-CoV-2 infection.

Although autophagy aims at clearance, in an ongoing arm race, viruses have evolved the potent strategy to escape and inhibit autophagy for their benefit (Choi et al., 2018; Mao et al., 2019). To further investigate whether SARS-CoV-2 interferes with host cell mitophagy, we examined Hsp60 expression to reflect mitochondrial number. In Vero E6 cells, the abundance of Hsp60 remains constant post SARS-CoV-2 infection, indicative of unimplemented mitochondrial clearance (Figure 6A).

In mitophagy, flawed mitochondria were selectively sequestered into autophagosomes and subsequently degraded in lysosomes for mitochondria quality control (Lemasters, 2005; Ashrafi and Schwarz, 2013). We next performed immunofluorescent staining using antibodies against Hsp60, LC3 and Lamp2b, to explore the different stages in SARS-CoV-2-induced mitophagy. No apparent overlapping signals of Hsp60 and the autophagosome marker LC3, as well as Hsp60 and Lamp2b, were observed in Vero E6 cells at 12 h post SARS-CoV-2 infection (Figure 6B). These results suggesting that the flawed mitochondria after SARS-CoV-2 infection were not encapsulated by autophagosomes, as well as subsequently transported into lysosomes.

Finally, in order to find out the way in which SARS-CoV-2 inhibits mitophagy, we detected the binding ability of p62 to LC3 protein by immunoprecipitation. P62 contains many functional domains, of which UBA domain can bind to ubiquitinated substrate, while LIR domain can bind to LC3 protein, which provides a link for autophagosomes to selectively encapsulate ubiquitinated substrates (Ashrafi and Schwarz, 2013). We found that the binding ability of p62 to LC3 protein in Vero E6 cells decreased at 12 h post SARS-CoV-2 infection (Figure 6C), suggesting that SARS-CoV-2 hindered the binding of p62 to LC3 protein, thus inhibiting the p62-labeled mitochondria to be encapsulated by autophagosomes.

## Discussion

**FIGURE 7:**
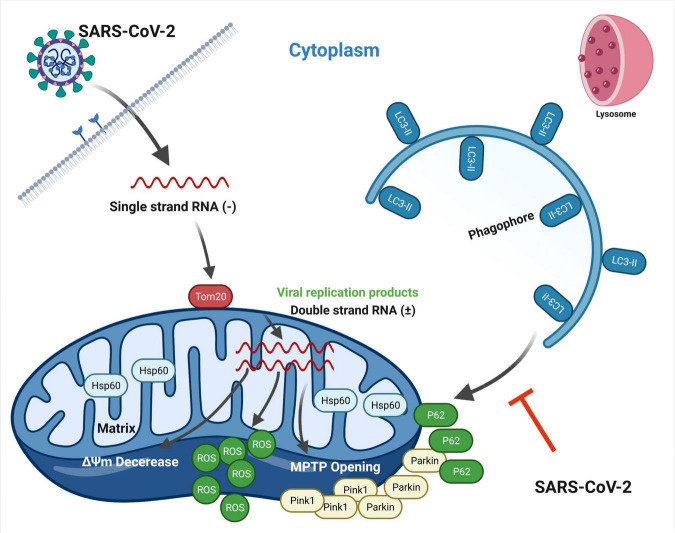
Schematic representation showing SARS-CoV-2-induced disruption of mitochondrial homeostasis. Upon infection, SARS-CoV-2 releases single strand RNA known to replicate in DMV structures. Tom20 facilitates the entry process of viral RNA into mitochondria, resulting in mitochondrial dysfunction, including the loss of ΔΨm, MPTP opening and increased ROS release. Concomitantly, mitophagy is initiated through Pink1/Parkin pathway by host cell for mitochondrial quality control and virus clearance. However, SARS-CoV-2 hinders the binding of p62 to LC3 protein, thus inhibiting the p62-labeled mitochondria to be encapsulated by autophagosomes. Mitophagy stays in the early stage. This figure is created with BioRender.com.

In this article, we present a model that SARS-CoV-2 infection disrupts mitochondrial homeostasis for their benefits (Figure 7). As the intermedium products of viral replication, the existence of SARS-CoV-2 dsRNA in mitochondria might be the match that set off these abnormities (Figure 1). This subcellular localization of SARS-CoV-2 dsRNA is consistent with a previous RNA-GPS study predicting the SARS-CoV-2 RNA genome and sgRNAs to be enriched toward the host mitochondrial matrix and nucleolus (Wu et al., 2020). Nevertheless, we do not know whether the dsRNA originates in mitochondria from SARS-CoV-2 replication, or comes from outside the mitochondria, such as DMV known as the site where β coronavirus replicates. To elucidate this, further studies are needed to examine if viral replication enzymes or viral particles exist in mitochondria. Importantly, the mitochondrial membrane stabilizer mdivi-1 (5 μM) and cyclosporin A (40 μM) reduced viral load at 24 and 48 h post SARS-CoV-2 infection (Figure 3), indicating a close relationship between SARS-CoV-2 replication and mitochondrial homeostasis. In transportation of SARS-CoV-2 RNA into mitochondria, Tom20 may play a pivotal role. Knockdown of Tom20 decreases mitochondrial damage caused by SARS-CoV-2, mitochondrial localization of dsRNA, and viral replication (Figure 4). These results are in line with previous studies, showing the deletion of Tom20 significantly reduced the association of many mRNAs with the mitochondria (Eliyahu et al., 2010; Lesnik et al., 2015).

In this context, mitochondrial dysfunction occurs. We observed the loss of ΔΨm, MPTP opening and increased ROS release along with time post SARS-CoV-2 infection (Figure 2). In response to mitochondrial damage, mitophagy was triggered by host cell through Pink1/Parkin pathway in order to maintain mitochondrial homeostasis and degrade viral RNA (Figure 5). However, SARS-CoV-2 hinders this process by inhibiting the binding of p62 to LC3 protein (Figure 6). Importantly, future studies are needed to elucidate the mechanisms by which SARS-CoV-2 restrains P62 and LC3 binding, and if a certain viral protein is responsible for this process.

In summary, the present study indicates an important role of Tom20 in the mitochondrial localization of SARS-CoV-2 RNA. Mitochondria might be closely associated with SARS-CoV-2 replication and in the cross fire of SARS-CoV-2 and host cell defense. SARS-CoV-2 causes mitochondrial dysfunction, and by inhibiting the binding of p62 and LC3, the virus blocks mitophagy that aims at mitochondrial quality control. This study will increase our understandings of the interaction between SARS-CoV-2 and the host cell, and assist the development of effective interventions targeting mitochondria.

## Materials and Methods

### Cell Lines, Viruses and Chemicals

African green monkey kidney cell Vero E6 (ATCC, CRL-1586) and human hepatocarcinoma cell Huh-7 (JCRB, 0403) were maintained in Dulbecco’s modified Eagle medium (DMEM) containing 10% fetal bovine serum (FBS), supplemented with 50 U ml–1 of penicillin and 50 μg ml–1 of streptomycin.

The SARS-CoV-2 strain BetaCoV/Beijing/IME-BJ01/2020 was originally isolated by CFQ’s laboratory as described previously (Gordon et al., 2020; Shang et al., 2021a,b). SARS-CoV-2 stocks were propagated and titered on Vero E6 cells. All work with live virus was performed in ABSL-3 in Changchun Veterinary Research Institute. In our experiments, cells were infected with SARS-CoV-2 at a MOI of 0.008.

Mdivi-1 (HY-15886) and cyclosporin A (HY-B0579) were purchased from MedChemExpress (New Jersey, United States).

### Antibodies

The rabbit or mouse antibodies against GAPDH (5174), Hsp60 (12165), Calnexin (2679) and LC3 (83506) were obtained from Cell Signaling Technology (Danvers, MA, United States). The rabbit antibody against LC3 (L8918) was obtained from Sigma-Aldrich. The rabbit antibody against Tom20 (ab186735) and Anti-SARS-CoV-2 nucleocapsid protein antibody (ab271180) were purchased from Abcam (Cambridge, United Kingdom). The rabbit or mouse antibodies against Pink1 (23274-1-AP), Parkin (66674-1-Ig), HSP60 (66041-1-Ig), Lamp2b (27823-1-AP or 66301-1-Ig) and P62 (66184-1-Ig) were obtained from Proteintech (Chicago, United States). The mouse anti-dsRNA (10010200) antibody was obtained from SCICONS (Szirák, Hungary). The Alexa Fluor^®^ 647-conjugated anti-mouse secondary antibody (4410), Alexa Fluor^®^ 488-conjugated anti-rabbit secondary antibody (4412) and horseradish peroxidase (HRP)–conjugated secondary antibodies (7074 or 7076) were purchased from Cell Signaling Technology. The FITC-conjugated anti-mouse secondary antibody (A0568) and Cy3-conjugated anti-rabbit antibody were purchased from Beyotime Biotechnology (Shanghai, China). The10-nm colloidal gold-conjugated secondary antibodies (G7777) was purchased from Sigma-Aldrich.

### siRNA Transfection

The control siRNA (sc-37007), siRNA against Tom20 (sc-36691) and siRNA against Pink1 (sc-44598) were purchased from Santa Cruz Biotechnology (California, United States). Twenty-four hours after seeding, the Vero E6 cells were transfected with 50 nM of siRNA using Lipofectamine™ RNAiMAX reagent (Thermo Fisher Scientific, 13778150) and experiments were conducted 48 h post transfection.

### Immunofluorescence Analysis

SARS-CoV-2-infected Vero E6 or Huh-7 cells were cultured for an indicated time on coverslips. Then, cells were fixed with 4% paraformaldehyde for 1 h and blocked with 0.3% Triton X-100 and 5% Bovine Serum Albumin (BSA) for 1 h. After washing for three times with PBS, specific primary antibodies were added and incubated overnight at 4°C. Cells were then washed and incubated with the anti-rabbit or anti-mouse fluorescent secondary antibodies for 1 h. Images were captured using a fluorescence microscope (Olympus BX43).

### Immunoelectron Microscopy

SARS-CoV-2-infected Vero E6 cells were fixed with ice-cold 4% paraformaldehyde containing 0.5% glutaraldehyde for 4 h. The fixed cells were washed with PBS for three times and passed through the following procedures: agarose solidification, dehydrate, resin penetration, embedding and polymerization. The blocks were cut to 70–80 nm ultrathin sections and immunolabeled with anti-dsRNA antibodies on the nickel grids, followed by incubation with 10-nm colloidal gold-conjugated goat anti-mouse antibodies. Images were captured under a transmission electron microscope (TEM).

### Analysis of Mitochondrial Function

Mitochondrial membrane potential (ΔΨm) was detected by JC-1 staining. Vero E6 and Huh-7 cells were seeded on coverslips at a density of 1 × 10^5^ cells/well in 12-well plates. Mock- or SARS-CoV-2-infected Vero E6 and Huh-7 cells were cultured for an indicated time. At 3, 6, and 12 h post infection, cells were washed with PBS for three times. 10 μg/ml JC-1 fluorescent dye (Sigma Aldrich, T4069) was added and incubated for 15 min. Images were captured by fluorescence microscopy.

Mitochondria permeability transition pore (MPTP) opening was assessed by the tetramethylrhodamine methyl ester (TMRM) probe (Sigma Aldrich; T5428). At 3, 6, and 12 h post infection, Vero E6 and Huh-7 cells were washed with PBS for three times. 300 nM TMRM was added and incubated for 15 min. Images were captured by fluorescence microscopy.

Reactive Oxygen Species (ROS) release was detected by the carboxy-H2DCFDA probe (Invitrogen, I36007). At 3, 6, and 12 h post infection, Vero E6 and Huh-7 cells were washed with PBS for three times. 25 μM carboxy-H2DCFDA working solution were added and incubated for 30 min. Cells were then returned to pre-warmed growth medium and incubate at 37°C for another 1 h. Images were captured by fluorescence microscopy. After the above steps, flow cytometry can also be performed to observe the changes in ROS levels.

### Crystal Violet Staining

Vero E6 cells were seed to a 6-well plate and cultured for 24 h. Then the SARS-CoV-2 infection was performed. After 48 h, the supernatant was discarded, and washed with PBS for three times. The crystal violet staining solution was added 600 μL to each well, and the cells were stained for 10 min at room temperature. Finally, the cells were washed with PBS for three times, and images were captured for analysis.

### Transmission Electron Microscope

Vero E6 cells were mock-infected or SARS-CoV-2-infected for 24 h and then fixed with 2.5% glutaraldehyde. Areas containing cells were block mounted and sliced. Ultrathin sections were contrasted with uranyl acetate and lead hydroxide, and subsequently viewed under a TEM (Hitachi, HT7800).

### Immunoblotting Analysis

Cells were seeded in 6-well plates and infected with SARS-CoV-2 after incubation for 24 h. At various time points as indicated, cells were collected, lysed and protein concentration was determined by BCA protein assay kit (Beyotime, P0010). After mixed with loading buffer and boiled for denaturing, equal amounts (30 μg) of proteins were electrophoresed, transferred into a membrane and then incubated overnight at 4°C with specific primary antibodies. After further incubation with HRP-conjugated secondary antibodies, the specific bands were visualized with the Pierce ECL Immunoblotting Substrate (Thermo Fisher Scientific, 32106) using the Amersham Imager 600 system. In siRNA gene silencing experiments, cells were collected without SARS-CoV-2 infection and subjected to the immunoblotting procedure described above.

### Real-Time Quantitative PCR Analysis

Vero E6 cells were seeded in 12-well plates at a density of 2 × 10^5^ cells/well. After SARS-CoV-2 infection, cells were incubated for an indicated time. Viral RNA in supernatant was isolated with the QIAamp Viral RNA Kit (Qiagen, 52906) following the manufacturer’s instructions. Virus copies were then detected by RT-qPCR methods with the HiScript II One Step RT-qPCR SYBR Green Kit (Vazyme Biotech, Nanjing, China) using an ABI 7500 real time PCR system (Applied Biosystems, CA, United States). The protocol for RT-qPCR was as follows: 50°C for 15 min, 95°C for 30 s, followed by 45 cycles at 95°C for 10 s and 63°C for 35 s. The specific primers used to detect the SARS-CoV-2 N gene were as follows: Forward: 5′-GGGGAACTTCTCCTGCTAGAAT-3′; Reverse: 5′-CAGACATTTTGCTCTCAAGCT-3′. The sequence of Taqman probe was as follows: 5′-FAM-TTGCTGCTGCTTGACAGATT-TAMRA-3′.

### Co-immunoprecipitation Assays

Mock- or SARS-CoV-2-infected Vero E6 cells were cultured for 12 h. At least 2 × 10^7^ cells were collected, lysed and protein concentration was determined by BCA protein assay kit (Beyotime, P0010). Some samples were denatured and subjected to immunoblotting procedure for input experiments. The rest of the samples were incubated with anti-P62 or IgG for 4 h at 4°C, followed by incubation with protein A-agarose for another 2 h at 4°C. The bound proteins were eluted with 1 × SDS loading buffer, denatured and analyzed by immunoblotting with anti-LC3 antibodies.

### Statistical Analysis

Statistical significance was determined using GraphPad Prism, Version 8.0 (GraphPad Software, San Diego, CA, United States). Data were presented as mean ± SEM in all experiments and analyzed with *t* test. *p* < 0.05 was considered statistically significant.

## Data Availability Statement

The original contributions presented in the study are included in the article/supplementary material, further inquiries can be directed to the corresponding author/s.

## Ethics Statement

The animal study was reviewed and approved by Animal Welfare and Ethics Committee of the Veterinary Institute at the Academy of Military Medical Sciences.

## Author Contributions

XL, YL, MT, and NJ conceived and designed the animal experiments. CS and ZL performed the experiments and wrote the manuscript. YZ, JL, CG, CZ, and NL performed the experiments. CS analyzed the data. XL and YL revised the manuscript. All authors have read and approved the final manuscript.

## Conflict of Interest

The authors declare that the research was conducted in the absence of any commercial or financial relationships that could be construed as a potential conflict of interest.

## Publisher’s Note

All claims expressed in this article are solely those of the authors and do not necessarily represent those of their affiliated organizations, or those of the publisher, the editors and the reviewers. Any product that may be evaluated in this article, or claim that may be made by its manufacturer, is not guaranteed or endorsed by the publisher.
